# Quantitative structure activity relationship and risk analysis of some pesticides in the goat milk

**DOI:** 10.1186/1735-2746-10-4

**Published:** 2013-01-04

**Authors:** Faqir Muhammad, Mian Muhammad Awais, Masood Akhtar, Muhammad Irfan Anwar

**Affiliations:** 1 Department of Physiology and Pharmacology, University of Agriculture, Faisalabad 38040, Pakistan; 2 Department of Pathobiology, College of Veterinary and Animal Sciences, Jhang, Sub campus University of Veterinary and Animal Sciences, Lahore, Pakistan; 3 Department of Parasitology, University of Agriculture, Faisalabad, 38040, Pakistan; 4 Poultry Research Institute, Office of Deputy District Livestock Officer (Poultry), Faisalabad, Pakistan

**Keywords:** Goat milk, Pesticides, Residues, QSAR model

## Abstract

The detection and quantification of different pesticides in the goat milk samples collected from different localities of Faisalabad, Pakistan was performed by HPLC using solid phase microextraction. The analysis showed that about 50% milk samples were contaminated with pesticides. The mean±SEM levels (ppm) of cyhalothrin, endosulfan, chlorpyrifos and cypermethrin were 0.34±0.007, 0.063±0.002, 0.034±0.002 and 0.092±0.002, respectively; whereas, methyl parathion was not detected in any of the analyzed samples. Quantitative structure activity relationship (QSAR) models were suggested to predict the residues of unknown pesticides in the goat milk using their known physicochemical characteristics including molecular weight (MW), melting point (MP), and log octanol to water partition coefficient (Ko/w) in relation to the characteristics such as pH, % fat, specific gravity and refractive index of goat milk. The analysis revealed good correlation coefficient (R^2^ = 0.985) for goat QSAR model. The coefficients for Ko/w and refractive index for the studied pesticides were higher in goat milk. This suggests that these are better determinants for pesticide residue prediction in the milk of these animals. Based upon the determined pesticide residues and their provisional tolerable daily intakes, risk analysis was also conducted which showed that daily intake levels of pesticide residues including cyhalothrin, chlorpyrifos and cypermethrin in present study are 2.68, 5.19 and 2.71 times higher, respectively in the goat milk. This intake of pesticide contaminated milk might pose health hazards to humans in this locality.

## Introduction

Quantitative Structure Activity relationship (QSAR) refers to the mathematical expressions which interrelate the structural and physicochemical characteristics of a series of compounds with their biological activities [1]. QSAR models may be useful for estimating activities of any compound and predicting structures of high activity [2]. The idea behind this relationship is that biological activity is a function of chemical structures that can be described by molecular or physicochemical variables such as molecular weight, hydrophobicity and steric properties etc. [3]. Previously, different QSAR models have been developed to predict the activities of untested chemicals in drug discovery and toxicology [4,5] and in this regard our research group has also developed QSAR models for the prediction of heavy metal residues in the cattle milk [6] and pesticide residues in cattle milk cattle [7].

Widespread occurrence of any foreign chemical or toxicant in the environment is a matter of public health concern. Pesticides are widely used to increase the agricultural productivity by preventing the losses due to pests. Moreover, health departments also use these chemicals for controlling various insects having vector role in spreading the diseases like malaria, dengue fever and plague, etc. [8,9]. Along with the beneficial activities, these pesticides may also pose health hazards by contaminating the environment including both the atmosphere in which we breath and food chain. Many pesticides and their residues have been reported as contributory factors in several diseases such as heart diseases, cancers, Alzheimer’s disease and Parkinsonism [10,11]. Pesticide residues in feed and fodders may transfer into herbivores through food chain and assimilate into the body systems of the animal [12].

Due to the lipophilic nature of these pesticides, milk and other fat-rich substances are the key items for their accumulation. These toxicants get into the human body through the food chain, and cause serious health problems. After ingestion, lipophilic pesticides get absorbed from the intestine into the general circulation. Highly lipid soluble pesticides tend to concentrate in tissues with higher lipid contents including adipose tissue, brain, liver, kidneys and in milk [11].

Milk and other dairy products are commonly used commodities in almost all the societies of the world and have a pivotal role in human nutrition. The occurrence of pesticides residues in the milk is a matter of public health concern, so, it is very important to ensure the quality of milk with respect to pesticide residues. In this regard, most of the developed countries have established their maximum residue levels (MRLs) for pesticides in the milk and other dairy products.

In Pakistan, pesticides are pre-dominantly used in the provinces of Punjab and Sindh. Some pesticides are biodegradable while others persist in the soils for longer times [13]. Under local conditions, pesticide residues have been detected in different vegetables in Karachi [14], in fruits and vegetable in Islamabad [15] and in various tissues of fish in the local lakes [16]. In order to avoid the toxic health hazards, it is necessary to determine the levels of pesticides in edible tissues such as milk of common food animals such as goat and cattle that are reared on pesticides spraying areas.

Keeping in view the above mentioned scenerio, this study was designed to determine the residues of most commonly used pesticides (cyhalothrin, endosulfan, chlorpyrifos, cypermethrin and methyl parathion) in the milk of goat and to develop QSAR models of pesticides residues in the goat milk using the milk characteristics and physico-chemical properties of pesticides. These QSAR models will be useful for prediction of different pesticide residues in goat milk samples especially in developing countries like Pakistan, where experimental or observed values are not available or difficult to determine due to lack of instrumentation and funding constraints.

## Materials and methods

### Milk sampling

The milk samples were collected randomly from different villages situated within a radius of 25–35 km on two different localities (east and west) of Faisalabad city, Pakistan. A total of 100 samples were collected during the winter (mid Nov-mid Feb) and spring (mid Feb-mid April) seasons with a frequency of 10 samples/locality/month and stored in 25 mL clean sterilized glass bottles. All the samples were kept at −20°C till further analysis.

### Analysis of milk samples collected from different locations for their physico-chemical characteristics

All the milk samples collected from different localities were analyzed for their physico-chemical properties including pH, fat (%), specific gravity and refractive index to develop QSAR models between these physicochemical properties and the determined levels of pesticides residues in different milk samples. The pH of the milk samples were determined with a digital pH meter, specific gravity with lactometer, fat (%) by Gerber’s butyrometer and refractive index with the help of a refractometer at a wavelength of 589 nm.

### Sample preparation/analytical procedure

The five different pesticide species (cyhalothrin, endosulfan, chlorpyrifos, cypermethrin and methyl parathion) were analyzed for their detection followed by quantification in the milk samples of goat by following the methodology of Cardeal and Clauda [17] with slight modifications. Briefly, milk samples were prepared for analysis by mixing with a buffer solution (acetic acid and sodium acetate pH (4.5) with a 1:9 ratio (v/v) that resulted in the precipitation of milk proteins. All the samples were centrifuged and from each sample a 16 mL aliquot of the upper layer was transferred to a 20 mL head space vial. A manual solid phase micro extraction (SPME) holder along with 100 μm thickness polydimethylsiloxane (PDMS) fiber assembly was purchased from Supelco (Bellefonte, PA, USA) and pure pesticides [Cyhalothrin (PS-2018); Endosulfan (PS 81); Chlorpyrifos (PS 674); Cypermethrine (PS 1068) and Methyl parathion (PS 94)] were purchased from Chem Service, West Chester, PA USA. The fiber was immersed directly into the glass vial containing the sample for half an hour with continuous stirring. The fiber was retracted and immersed into methanol for elution of residues.

### High performance liquid chromatography (HPLC) analysis of pesticide residues in different milk samples

The eluted methanol was injected in to HPLC system (Shimadzu) equipped with UV–vis detector for analyses of pesticides. Thermohypersil-C_18_ column was used for analysis and a wavelength of 235 nm was used to get absorption spectra. A mixture of acetonitrile and methanol in a ratio (11:9 v/v%) was used as mobile phase and injection volume taken for each of the samples and standards for analysis was 20 μl.

### Statistical analysis

The quantitative data on the pesticide concentrations in milk samples was subjected to one way analysis of variance (ANOVA) and results were expressed as mean ±SEM. Multiple regression analysis was conducted on experimentally determined pesticide concentrations in the milk and data of physical parameters of both milk and pesticides using least sum of squares on Microsoft Excel version 2007. A QSAR model was suggested to calculate the concentrations of unknown pesticides in the milk using their known physico-chemical properties. Furthermore, other QSAR models were suggested to predict the concentrations of these pesticides using the milk characteristics of goat in the study area. Risk analysis was computed based upon the determined pesticide residues in the goat milk and provisional tolerable daily intakes of these pesticides as established by the regulatory authorities.

## Results

Milk samples of goat collected from different localities of Faisalabad were analyzed for the qualitative and quantitative detection of five different pesticides (cyhalothrin, endosulfan, chlorpyrifos, cypermethrin and methyl parathion). The residue analysis revealed that about 50% milk samples were contaminated with pesticides.

**Figure 1:**
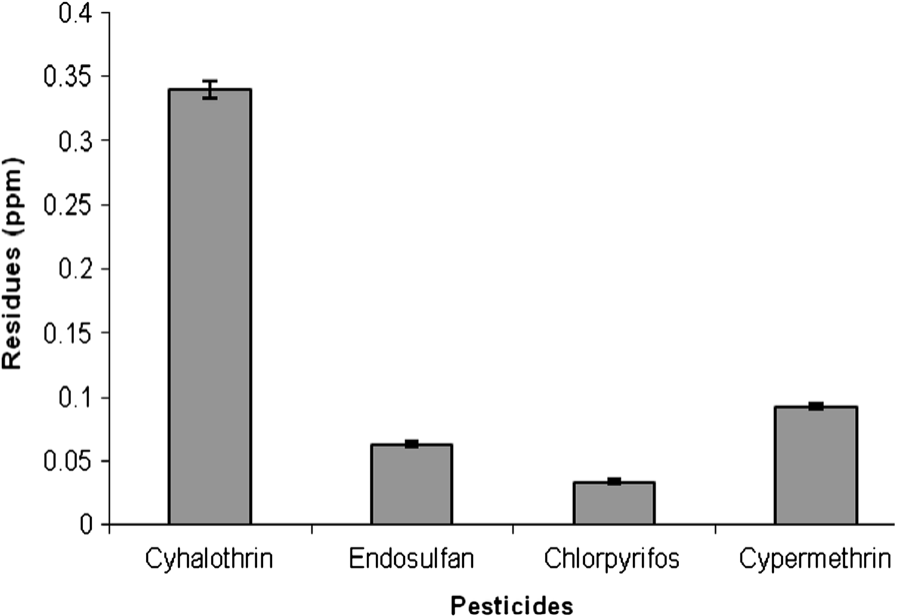
Mean ± SEM values (ppm) of pesticides in the goat milk collected from Faisalabad district of Pakistan.

Results of quantitative HPLC analysis (mean±SEM) of milk samples for pesticide residues are presented in the Figure 1. This figure shows that mean±SEM levels (ppm) of cyhalothrin, endosulfan, chlorpyrifos and cypermethrin are 0.34±0.007, 0.063±0.002, 0.034±0.002 and 0.092±0.002, respectively; whereas, methyl parathion was not detected in any of the analyzed samples. The levels of all the detected pesticide species under study were found to be exceeding maximum residual limits (MRL) as recommended by USFDA (Table 1) except endosulfan that could not surpass the MRL.

**Table 1:** Physico-chemical properties and maximum Residue Limits (MRLs) for pesticides established by World Health Organization

**Pesticides**	**Molecular weight (MW)**	**Melting point (°C)**	**Ko/w**	**Maximum Residual Limits (ppm)**
Cyhalothrin	450	10	6.90	0.2
Endosulfan	407	106	3.58	0.5
Chlorpyrifos	351	41	5.00	0.01
Cypermethrin	416	70	6.30	0.05

From all the pesticides under study, the occurrence of cypermethrin residues exceeding MLR was highest (25%; n=25/100) followed by cyhalothrin (21%; n=21/100) and chlorpyrifos (20%; n=20/100); whereas the endosulfan was under the MRLs in all the milk samples examined.

To study the relationship of residue levels of these pesticides with their own physico-chemical properties including molecular weight (MW), melting point (MP) and log octanol to water partition coefficient (Ko/w) (Table 1) and milk characteristics such as pH, fat(%), specific gravity and refractive index (Table 2) multiple regression analysis was conducted at a significance level of 0.05. The analysis revealed an excellent correlation coefficient (*R*^*2*^=0.985) for QSAR model that depicts a good prediction power of this model.

**Table 2:** Milk characteristics (mean values) of goat milk collected from different sites of Faisalabad-Pakistan

**Sample collection sites**	**Milk pH**	**Fat (%)**	**Specific gravity**	**Refractive index**
1	6.57	5.8	1.003	1.3489
2	6.49	6.2	0.998	1.3494
3	6.47	6.1	1.005	1.3471
4	6.56	6.0	0.998	1.3504
5	6.65	6.2	0.977	1.3474

### QSAR model

(1) LogKr=C+αMW+βMP+γKo/w

Where, intercept C = 0.00, MW coefficient *α* = 0.0049, MP coefficient *β* = −0.0039, and Ko/w coefficient *γ* = −0.258.

The coefficients of this QSAR model show that residue levels in milk are directly proportional to MW while inversely proportional to MP and Ko/w of pesticides.

Multiple regression equation and coefficients for pesticide residues in goat milk with respect to milk characteristics are presented in Table 3. The correlation coefficients (*R*^*2*^) for cyhalothrin, endosulfan, chlorpyrifos and cypermethrin residues in QSAR models were 0.996, 0.988, 0.996 and 0.998, respectively. Results showed a positive correlation of all the pesticide residues under study with respect to the pH (indicated by α) and % fat (indicated by β). Similarly, all the pesticide residues except Cyhalothrin showed a positive correlation with respect to specific gravity (indicated by *γ*). On the other hand, all the pesticide residues showed a strongly negative correlation with the refractive index (indicated by δ) that is depicted by higher values of δ as compared to the coefficients of all other milk characteristics.

**Table 3:** Multiple regression equation and coefficients for pesticide residues determined in the goat milk samples with respect to the milk characteristics

**Log Kr = C + α(pH) + β(% fat) + γ(specific gravity) + δ(refractive index)**					
**Pesticide**	***R***^***2***^	***α***	***β***	***γ***	**δ**
Cyhalothrin	0.996	0.1296	0.1231	−0.3786	−0.6529
Endosulfan	0.988	0.1465	0.0445	0.5307	−1.2570
Chlorpyrifos	0.996	0.1934	0.0648	0.9171	−1.8831
Cypermethrin	0.998	0.0519	0.0459	0.2058	−0.5421

Intercept C=0.

According to Food and Drug Administration, the daily recommended milk requirement for a healthy adult individual is 1.5 Kg [18]. Based upon which daily intake levels of pesticide residues including cyhalothrin, chlorpyrifos and cypermethrin in present study are 2.68, 5.19, 2.71 times higher, respectively in goat milk. Since endosulfan did not surpass the MRL so risk assessment for this pesticide was not calculated.

## Discussion

Pesticides are one of the major sources of contamination of dairy products due to the presence of their residues in animal feedstuffs. Other contributory factors, in this regard, may include the application of pesticides on farm animals, environmental contamination and accidental spills. Milk contamination may be avoided by hindering the entry of pesticides residues into the dairy animals through contaminated feedstuffs/food chain.

In the current study, residues analysis showed high levels of cyhalothrin, chlorpyrifos and cypermethrin in the goat milk samples exceeding MRLs. The result of our findings are comparable to those determined by Wong and Lee [19] who analyzed the milk samples collected from local market of Hong-Kong for organo-chlorine pesticides and reported that 42 out of 252 (16%) samples exceeded the level prescribed by codex committee on pesticides.

The percentage of milk samples contaminated with pesticide residues in present study was lower as compared to those reported by [14] who analyzed the 206 samples of different vegetables from Karachi, out of which 63% samples were found to be contaminated while 46% samples exceeded the MRL. At the same time, percentage (50%) of milk samples contaminated with pesticide residues was also lower than different food commodities (60%) as reported by [15,20]. Higher percentage of contaminated food commodity (fruits and vegetables) as compared to milk might be due to the direct exposure of these food items to pesticides. Regarding QSAR model, multiple regression equation 1 suggested that molecular weight, melting point and Ko/w were main determinants of pesticide residues in the goat milk. The equation 1 indicated a direct dependence of residue levels in milk on molecular weight and inverse dependence on melting point and Ko/w.

The residues of all the pesticides under study showed a positive correlation with respect to pH and % fat present in the milk samples (Table 3). This finding is in accordance with some previous studies [21,22] which reported the accumulation of a high level of pesticide residues in high fat milk as compared to low fat milk. Yang et al. [23] and Sheng et al. [24] showed that different pesticides show higher solubility at lower pH (acidic) level and this is consistent with the findings of the current study. Further, a similar dependence pattern of all the pesticide residues under study except cyhalothrin was observed for specific gravity and specific gravity has been regarded as a good determinant for prediction of heavy metal residues in the milk cattle and goat [6]. Earlier, no study has been conducted on the correlation of pesticide residues in the milk sample with its refractive index; however, present study showed a very strong negative correlation of all the pesticide residues with the refractive index depicted by higher refractive index coefficient values (Table 3). Therefore, refractive index may be considered as one of the most important determinants in this regard for the prediction of pesticide residues in the goat milk. The variable pattern of pesticide residue dependence upon milk characteristics signifies the need to construct the region specific QSAR models for residue prediction in the goat milk.

The amount of a chemical that can be consumed daily for a lifetime in the practical certainty, on the basis of all known facts, that no harm will result is termed as acceptable daily intake (ADIs) [25] and the ADIs of cyhalothrin, endosulfan, chlorpyrifos and cypermethrin are 0.2, 0.5, 0.01 and 0.05 ppm, respectively (Table 1) [26]. With respect to Food and Drug Administration recommendations, the daily intake levels of pesticide residues including cyhalothrin, chlorpyrifos and cypermethrin in present study are much higher in the goat milk which may pose a great threat to the infants and children in which goat milk is preferably used to nourish them for their better growth. Moreover, now a day, goat milk is extremely used in the cosmetics industry in the manufacturing of skin whitening soaps, creams, body lotions, shampoos, hair conditioners and after shave lotions, which are marketed in many countries such as US and Switzerland [27]. Being, lipophilic in nature, pesticides if present in the milk can easily penetrate into the skin and may lead to skin toxicity [28].

## Conclusions and recommendations

The findings of the present study indicated that 50% milk samples of goats were contaminated with pesticides and 20-25% samples of goats surpassed the MRL levels for different pesticides. QSAR models suggested can be successfully used in predicting the unknown pesticide residue levels in the milk by using their known physicochemical and milk characteristics. Risk analysis revealed that daily intake levels of pesticide residues including cyhalothrin, chlorpyrifos and cypermethrin in present study are higher that the recommended permissible levels that pose a great threat to the end consumers. Nevertheless, it is indispensible to monitor the presence of these compounds for their relevant implication in health care system and assess their levels in other zones of the country to shape a major representative panorama. Moreover, these findings suggest creating awareness in the farmer community and general public regarding the avoidance of pesticide residues in milk.

## Competing interests

The authors declare that they have no competing interests.

## Authors’ contributions

FM, the first author of this manuscript was involved in the analytical part of this experimental work. FM and MMA both were involved in statistical analysis and drafting of this manuscript. FM, MMA, MA and MIA were involved in the work plan and in the discussion of the results. MA and MIA read and corrected the manuscript. All authors read and approved the final manuscript.
